# Reflections on Long-Term Dental Outreach: Insights From Stakeholders in Rural Australia

**DOI:** 10.1177/23743735251383235

**Published:** 2025-10-07

**Authors:** Aiko Nagae, Lydia See, Alice Lu, Connie Cai Ru Gan

**Affiliations:** 1 School of Medicine and Dentistry, 5723 Griffith University, Southport, Australia; 2 School of Dentistry, 1974 University of Queensland, Brisbane, Australia; 3 Buddhist Compassion Relief Tzu Chi Foundation Australia, Brisbane, Australia

**Keywords:** access to care, patient experience, patient feedback, qualitative methods, oral health, social impact, voluntary dental care, dental outreach

## Abstract

Regional Australians experience poorer oral health compared to urban populations, with higher rates of tooth decay and gum disease. The National Oral Health Plan 2015–2024 addresses these challenges through dental outreach programs in isolated areas. While studies have clinical outcomes, the stakeholders’ experiences remain understudied. As such, this study aimed to explore stakeholders’ experiences of a 17-year-long-term annual dental outreach program in a regional Australian community. A total of 35 interviews were conducted. Findings from the thematic analysis showed that this outreach has improved the community's oral health outcomes and overall well-being. There were general sentiments about the project's positive impact beyond oral health, including boosting community morale and strengthening partnerships with local practitioners. Volunteers gained new perspectives that enhanced their personal and professional growth. Findings also suggested the need for interventions directed at preventative care and integrating other health screening and promotion activities, aiming to optimise outreach opportunities to address health disparities in resource-constrained areas.

## Introduction

Regional Australians experience poorer oral health compared to urban populations, with higher rates of tooth decay and gum disease.^1^ This disparity stems from limited preventative care due to accessibility to fluoridated water, higher product costs, and a dental workforce density 4.5 times lower than in urban areas.^1^ The National Oral Health Plan 2015–2024 highlights the importance of dental outreach programs in isolated areas to address these issues,^2^ by strengthening the limited organisations in Queensland providing such services.^3,4^

Given that these services are typically offered for only a few days to weeks per year, a previous study has raised concerns about their impacts on the community and sustainability.^5^ While existing research examined their impacts through patients’ oral health improvements^3,4^ and surveys on volunteer dental students,^6,7^ the experiences of patients, nonclinical volunteers, and local service providers remain understudied. Yet, exploring their perceptions is crucial to understanding the program's nuanced impacts on the wider community.^8^ This study aimed to explore the impact of program continuity on stakeholders’ experiences and perspectives on a dental outreach program. Using a qualitative approach, this study aims to identify potential areas of impact and inform the implementation of any future planning of oral health programs for priority populations.

## Methods

This study employed a qualitative method to explore the stakeholders’ perceptions and experiences of a dental outreach program in regional Australia. Ethical approval for the study was obtained from Griffith University's human ethics committee (GU Ref No. 2024/159). A convenience sampling method was used to collect initial insights for practicality and feasibility purposes. Tzu Chi volunteers (VTs) helped identify service recipients (SRs) and VTs based on their roles, locations, gender, and experiences ranging from first-time to multiple engagements. While this approach may have introduced selection bias, particularly toward individuals with existing connections to VTs, efforts were made to ensure a broad representation of experiences. The research team then recruited participants during the medical outreach, provided information about the study, and obtained participants’ written consent. To recruit local practitioners (LPs), the research team sent emails to contact relevant partners who were involved in the program. All interviews, except one online, were carried out in person during the medical outreach between 28 and 31 March 2024 by AN and CG. Questions included regarding participants’ demographics, feedback on treatments, and future improvements on outreach programs (see Supplemental Table 1). Each interview was audio-recorded and lasted from15 minutes to one hour. The interview results were de-identified and transcribed by AN using NVivo version 14. A thematic analysis developed by Braun and Clarke^9^ guided the data analysis method. This approach identifies repeated patterns within the data, which makes it appropriate for exploring stakeholders’ experiences and thoughts on medical outreach programs.^9^ IDs were assigned according to their roles: LPs, VTs, and SRs.

## Setting

The study was conducted in Tara, a rural town in Southwest Queensland.^10^ With no resident dentist, residents must drive over 100 km to access dental services in neighboring regions. To address this gap, the Tzu Chi Foundation (TCF) has provided free dental services since 2007. Alongside the Royal Flying Doctor Services, it remains one of the only providers of dental care in the community. Initially focusing on general dental services, the scope has expanded to include specialist dental, denture and allied health services.

## Results and Discussion

A total of 35 participants were interviewed for the study. The sample included 18 SRs, 13 VTs, and 4 LPs. Almost all SRs, except for two, received dental care, while allied health services and social services were provided to 10 SRs. VT and LP included both clinicians and nonclinicians (see Table 1).

**Table 1.** Summary of Demographics of the Interviewees.

	Volunteers *N* = 13 (37.2%)	Local Practitioners *N* = 4 (11.4%)	Service Recipients *N* = 18 (51.4%)
Sex			
Male	4 (30.8%)	2 (50%)	8 (44.4%)
Female	9 (69.2%)	2 (50%)	10 (55.6%)
Role			
Clinical	6 (46.2%)	2 (50%)	—
Nonclinical	7 (53.8%)	2 (50%)	—
Provided services^*^			
Dental care^a^	—	—	16 (88.9%)
Allied health^b,c^	—	—	7 (38.9%)
Social service^a,b^	—	—	3 (16.7%)

^a^ The number of service recipients who received allied health services and social services includes those who received dental treatment.

^b^ Allied health services included physiotherapy and acupuncture, and social services involved bushfire relief.

### The Profile of Tara

Since the COVID pandemic, the population in Tara has doubled in size due to lower living costs (LP4). However, the majority of residents have low socioeconomic status, with most relying on welfare: “There's a lot of very poor people living in Tara, it is not a wealthy town and in fact nearly 80% of the town my patients are on welfare recipients of one kind or another” (LP4). These situations, coupled with the lack of job opportunities, are perceived to contribute to drug issues and poor self-care and parenting: “People just don't look after themselves … I try and just do the best I can with my own children. But if they don't know how to look after their own teeth, how are they going to teach their children?” (SR13). Participants also described Tara as a neglected town: “It looks small but it's big, because it's a small town like this gets neglected very easily” (SR6). They observed this was due to a lack of access to healthcare. As of 2024, there is no resident dentist in town, making dental services inaccessible: “That's (travel to neighbouring town for dental appointments) only four-hour round trip. But I can't do that. That is an enormous round trip in one day” (SR11).

### Program Development and Perceived Impact Over Time

Since 2007, the program has reached capacity to provide services to “10% of the population in one weekend” (LP4). Although the population in Tara differs, ranging from 1980 (Google^11^) and 3851 in 2021 (census data^12^) to 5300 active patient records (from the local clinic), this sentiment indicates that this program has reached a significant population throughout the years. Additionally, the number of volunteers increased from 20 to 30 people to 140 volunteers (VT31). Most participants gave positive feedback about the program: “I’d never heard of any great, wonderful people coming here to do this” (SR21). Key enablers included the quality of treatment: “This experience is much softer with your mouth” (SR9) and the welcoming atmosphere: “They're so welcoming … they were there to listen and to hear what we had to say. There was no judgement” (SR11). Additionally, VTs mentioned the positive impact on oral health outcomes: “We hear less and less of that (complex and serious oral health conditions). So I do feel that we have brought a change to people's lives here” (VT31).

### Impact Beyond Oral Health Outcomes

Importantly, the interviews revealed that this program improved patients’ self-esteem: “It (denture) was never really a bit of plastic in my mouth. It was part of my identity” (SR6): “Once a patient gets the dental treatment completed, this gets rid of any pain and discomfort that they have in mouth. They're much more aware and also likely to gain to healthier habits” (LP35). These observations are supported by the previous findings,^13^ which show that dental disorders affect an individual's self-esteem.

Participants also shared that they felt valued and were attending the event even to meet the volunteers. The respondents who experienced a recent bushfire emphasised that the program not only provides dental services but also focuses on emotional support:

Their house was destroyed, absolutely destroyed … You guys are a godsend (the bushfire relief aid and medical services that Tzu Chi provides during the 2024 outreach program). And I mean that in the most polite way. People think that they'll never get the help out (in Tara) (SR27).

These nondental improvements suggest that this outreach may be addressing broader community issues. Evidence^14,15^ shows that improved self-esteem and social network are significantly associated with better oral health outcomes, indicating that this outreach could be positively influencing patients’ attitudes toward oral health.

### Continuity and Cycle of Positivity

Participants also expressed that both SRs and VTs share positivity:

“They might be having a bad day … It just cheers him (SR) up, just a little bit. And then maybe the next person might be the same and it's just rubs off just being nice, and that's what you guys are doing. It's not just about teeth and sticking needles” (SR6): “[Tzu Chi] people are very polite and you can see the way they speak, very soft … (I) get angry, you know. So I come here, I see people, even though in a very explosive environment, still very calm.” (VT19)

In the Australian context, the limited government resources for primary healthcare in the dental sector is pronounced, particularly in resource-constrained regional areas.^16^ Prioritising curative care rather than prevention is a common trend in dental outreach programs.^17,18^ However, study participants have highlighted the nondental benefits, including enhanced mental health and broader access to other health services. This program builds relationships between healthcare providers and underserved communities while promoting preventive care awareness and providing strong justification for continued investment in these initiatives. These findings highlighted the importance of continuity and trust in effectively engaging the community, which can inform the design of similar programs in other settings.

It can further contribute to the development and implementation of the national oral health plans for individuals in regional and remote areas, those who have experienced domestic violence, and those with mental health and medical complexities.

## Limitations

A couple of limitations should be addressed. First, there may have been selection bias as participants were recruited via VTs, who identified potential individuals based on the research team's selection criteria. Second, despite interviewers clarifying no TCF affiliation, some participants identified the interviewers as VTs, possibly leading to response bias. Last, since this study focused on qualitative data, the findings are subjective and cannot be generalised. To gain a more comprehensive understanding, a quantitative method should also be used to examine long-term impacts on the community, including VTs and LPs.

## Conclusion

This study aimed to explore stakeholders’ experiences with a dental outreach program in a regional area. The findings suggested that this program builds relationships between healthcare providers and community members while promoting preventive care awareness. While the feedback has been positive, there is a need for continuous evaluation to achieve the balance of curative and preventive care for long-term benefits.

## Supplemental Material

sj-docx-1-jpx-10.1177_23743735251383235 - Supplemental material for Reflections on Long-Term Dental Outreach: Insights From Stakeholders in Rural Australia Supplemental material, sj-docx-1-jpx-10.1177_23743735251383235 for Reflections on Long-Term Dental Outreach: Insights From Stakeholders in Rural Australia by Aiko Nagae, Lydia See, Alice Lu and Connie Cai Ru Gan in Journal of Patient Experience

sj-docx-2-jpx-10.1177_23743735251383235 - Supplemental material for Reflections on Long-Term Dental Outreach: Insights From Stakeholders in Rural Australia Supplemental material, sj-docx-2-jpx-10.1177_23743735251383235 for Reflections on Long-Term Dental Outreach: Insights From Stakeholders in Rural Australia by Aiko Nagae, Lydia See, Alice Lu and Connie Cai Ru Gan in Journal of Patient Experience

## References

[1] The Department of Health and Aged Care . *Healthy mouths, healthy lives – Australia’s National Oral Health Plan 2015–*2024. Updated May 8, 2023. Accessed January 15, 2025. https://www.health.gov.au/resources/publications/healthy-mouths-healthy-lives-australias-national-oral-health-plan-2015-2024?language=en

[2] Australian Institute of Health and Welfare . *Oral health and dental care in Australia* . 2023. Updated October 4, 2024. Accessed January 15, 2025. https://www.aihw.gov.au/reports/dental-oral-health/oral-health-and-dental-care-in-australia/contents/hospitalisations/potentially-preventable-hospitalisations

[3] Margolis SA Ypinazar VA . Aeromedical retrieval for critical clinical conditions: 12 years of experience with the Royal Flying Doctor Service, Queensland, Australia. J Emerg Med. 2009;36(4):363-368. doi:10.1016/j.jemermed.2008.02.057 18814993

[4] Gardiner FW Richardson A Gale L , et al. Rural and remote dental care: patient characteristics and health care provision. Aust J Rural Health. 2020;28(3):292-300. doi:10.1111/ajr.12631 32462697

[5] Holmgren C Benzian H . Dental volunteering - a time for reflection and a time for change. Br Dent J. 2011;210(11):513-516. doi:10.1038/sj.bdj.2011.426 21660012

[6] Suresan V Jnaneswar A Swati SP Jha K Goutham BS Kumar G . The impact of outreach programs on academics development, personal development and civic responsibilities of dental students in Bhubaneswar city. J Educ Health Promot. 2019;8(1):188-196. doi:10.4103/jehp.jehp_56_19 PMC6796316 31867373

[7] Bhayat A Mahrous MS . Impact of outreach activities at the College of Dentistry, Taibah University. J Taibah Univ Med Sci. 2012;7(1):19-22. doi:10.1016/j.jtumed.2012.07.008

[8] Walker H Pope J Morrison-Saunders A , et al. Identifying and promoting qualitative methods for impact assessment. Impact Assess Project Appraisal. 2024;42(3):294-305. doi:10.1080/14615517.2024.2369454

[9] Braun V Clarke V . Thematic analysis : A practical guide. Sage; 2022.

[10] Queensland Government . n.d. *Queensland globe*. Accessed July 27, 2024. https://qldglobe.information.qld.gov.au/

[11] Google . Google Maps. Accessed July 29, 2024. https://www.google.com/maps/@-27.2470849,149.8237403,10z?entry=ttu

[12] Australian Bureau Statistics . *Tara.* Accessed July 29, 2024. https://abs.gov.au/census/find-census-data/quickstats/2021/307011178

[13] Kaur P Singh S Mathur A , et al. Impact of dental disorders and its influence on self esteem levels among adolescents. J Clin Diagn Res. 2017;11(4):ZC05-ZC08. doi:10.7860/JCDR/2017/23362.9515 PMC5449896 28571250

[14] Neethu SS Manjunath PP Yashoda R . Association of social network and social support with oral health among institutionalized elderly: a cross-sectional study in Bangalore city. Clin Epidemiol Glob Health. 2023;24:101439. doi:10.1016/j.cegh.2023.101439

[15] Pazos CTC Austregésilo SC Goes P . Self-esteem and oral health behavior in adolescents. Cien Saude Colet. 2019;24(11):4083-4092. doi:10.1590/1413-812320182411.02492018 31664381

[16] Gamarra S Bärnighausen K Wachinger J , et al. We had to take a hammer to get some roots out’ - experiences, motivations and challenges among volunteer dentists: a qualitative study. Br Dent J. 2021; 231(11):681-686. doi:10.1038/s41415-021-3222-6 34381176

[17] Patel J Bear N Long R , et al. The Kimberley Dental Team: a process evaluation of a volunteer dental programme serving remote Aboriginal communities in Australia. Community Dent Oral Epidemiol. 2023;51(6):1241-1249. doi:10.1111/cdoe.12885 37306125

[18] Sloan AJ Wise SL Hopcraft M . Primary care dentistry: an Australian perspective. J Dent. 2024;145:104996. doi:10.1016/j.jdent.2024.104996 38621524

