# Supplementary material for: Reflections on Long-Term Dental Outreach: Insights From Stakeholders in Rural Australia
Source: J Patient Exp. 2025 Oct 7;12:23743735251383235. doi: 10.1177/23743735251383235 (PMC12504834; doi:10.1177/23743735251383235)
Supplement: sj-docx-1-jpx-10.1177_23743735251383235 - Supplemental material for Reflections on Long-Term Dental Outreach: Insights From Stakeholders in Rural Australia [file sj-docx-1-jpx-10.1177_23743735251383235.docx]

**Supplementary Table 1**: Detailed breakdown of interviewee demographics.

| **No.** | **Code** | **Sex** | **Volunteers/Local Practitioners** | **Service Recipients** |
| --- | --- | --- | --- | --- |
|  |  |  | **profession/role** | **Provided care** |
| 1 | LP | F | Non-clinical | Dental care |
| 2 | LP | F | Non-clinical | - |
| 3 | VT | F | Clinical | - |
| 4 | LP | M | Clinical | - |
| 5 | SR | F | - | Dental care |
| 6 | SR | M | - | Dental care |
| 7 | SR | F | - | Dental care |
| 8 | SR | F | - | Dental care, physiotherapy |
| 9 | SR | F | - | Dental care |
| 10 | VT | F | Clinical | - |
| 11 | SR | F | - | Acupuncture, bushfire relief |
| 12 | VT | F | Clinical | - |
| 13 | SR | F | - | Acupuncture |
| 14 | VT | F | Non-clinical | - |
| 15 | SR | F | - | Dental care |
| 16 | SR | F | - | Dental care |
| 17 | SR | M | - | Dental care, bushfire |
| 18 | SR | F | - | Dental care, acupuncture, bushfire |
| 19 | VT | M | Non-clinical | - |
| 20 | VT | F | Non-clinical | - |
| 21 | SR | M | - | Dental care |
| 22 | SR | M | - | Dental care, physiotherapy |
| 23 | SR | M | - | Dental care, physiotherapy |
| 24 | SR | F | - | Dental care, physiotherapy |
| 25 | VT | F | Clinical | - |
| 26 | SR | M | - | Dental care |
| 27 | SR | M | - | Dental care |
| 28 | VT | M | Non-clinical | - |
| 29 | VT | M | Clinical | - |
| 30 | VT | F | Non-clinical | - |
| 31 | VT | M | Non-clinical | - |
| 32 | SR | M | - | Dental care |
| 33 | VT | F | Non-clinical | - |
| 34 | VT | F | Clinical | - |
| 35 | SP | M | Clinical | - |
