# Supplementary material for: Reflections on Long-Term Dental Outreach: Insights From Stakeholders in Rural Australia
Source: J Patient Exp. 2025 Oct 7;12:23743735251383235. doi: 10.1177/23743735251383235 (PMC12504834; doi:10.1177/23743735251383235)
Supplement: sj-docx-2-jpx-10.1177_23743735251383235 - Supplemental material for Reflections on Long-Term Dental Outreach: Insights From Stakeholders in Rural Australia [file sj-docx-2-jpx-10.1177_23743735251383235.docx]

**Supplementary Table 2: Interview Questions**

| ***Stakeholder Type*** | **Questions** |
| --- | --- |
| Local Practitioners | What is your role in the Tara community? |
|  | What was your overall experience with attending the medical fairs? |
|  | What does social impact mean to you?   - What would this look like for you as an individual and as a town/community? |
|  | What kind of social impact would you be interested in seeing as a result of having annual medical fairs in Tara? |
|  | What does social impact look like to you immediately after the Tzu Chi annual medical fair event, and what will it mean to you long term?   - What does that look like in the immediate future after the Tzu Chi annual medical fair event? What about long-term? |
| Service recipients | How many medical fairs have you attended over the years? |
|  | What services did you access over the years? |
|  | What was your overall experience with attending the medical fairs?   - Was there anything in particular you enjoyed? Found useful? |
|  | What has receiving the treatments meant to you |
|  | How has being able to access these health services improved your quality of life? |
|  | If there were no Tara medical fairs, where would you go to access services? |
| Tzu Chi Volunteers | What is your role in the Tara Medical Fairs Outreach Program? What type of service/support do you provide? |
|  | What would social impact mean to you? |
|  | What would that look like for you in the immediate and long term? |
|  | What kind of social impact have you seen/experienced as a result of having annual medical fairs in Tara? |
|  | From attending this event, do you see it changing your perspective on your daily activities (for all volunteers) and clinical practice (for health professional volunteers)? |
|  | From being involved in this event, did you gain/learn anything from the experience?   - Differentiating questions: - From the perspective of being in the leadership/working committee role - From the perspective of a participant volunteer |
|  | For the organising committee, what are the health outcomes and social impact you would like to see from the annual Tara medical fairs?   - What kind of social impact would you be interested in seeing? |
